# The association between attitude, perceived norm, and perceived behavioral control with the provision of Clinical Work-Integrating Care: A reasoned action approach

**DOI:** 10.1016/j.pecinn.2025.100416

**Published:** 2025-06-27

**Authors:** Authors: Lana Kluit, Annechien Beumer, Coen A.M. van Bennekom, Angela G.E.M. de Boer, Astrid de Wind

**Affiliations:** a Amsterdam UMC, University of Amsterdam, Public and Occupational Health, Meibergdreef 9, Amsterdam, the Netherlands; b Amsterdam Public Health Research Institute, Societal Participation and Health, Amsterdam, the Netherlands; c Amphia Hospital, Upper Limb Unit Department of Orthopedic Surgery, Breda, the Netherlands; d Heliomare Rehabilitation Centre, Research and Development, Wijk aan Zee, the Netherlands; e Cancer Center Amsterdam, Cancer Treatment and Quality of Life, Amsterdam, the Netherlands

**Keywords:** Work participation, Clinical work-integrating care, Specialized medical care, Health care improvement, Return to work

## Abstract

**Background:**

Clinical Work-Integrating Care (CWIC) brings important attention to issues emerging from the interrelationship between health and work. Yet, for various reasons, CWIC is not routinely delivered in clinical healthcare. This study focuses on why medical specialists do or do not provide CWIC, applying a reasoned action approach.

**Objective:**

To examine the associations between attitude, perceived norm, and perceived behavioral control with the provision of CWIC.

**Methods:**

A cross-sectional survey was distributed to Dutch medical specialists. Multivariable regression analysis was used to investigate the associations between attitude, perceived norm, and perceived behavioral control on the ability to provide CWIC with the frequency of actual CWIC provision.

**Results:**

In total, 160 medical specialists completed the survey. The sample consisted of 12 surgical specialists (8 %), 113 non-surgical specialists (71 %), and 35 rehabilitation specialists (22 %). After adjustment for confounders, a favorable attitude was significantly associated with providing CWIC (*p* < .01), while perceived norm and perceived control were not (*p* = .74 and *p* = .85, respectively).

**Conclusions:**

Medical specialists who expressed a favorable attitude towards addressing work during consultations were more likely to provide CWIC. Thus, addressing specialists' attitudes is an important element to implementing CWIC.

## Highlights

• Clinical Work-Integrating Care (CWIC) focuses on the link between health and work.
• A favorable attitude towards CWIC is associated with increased provision of CWIC.
• Higher perception that CWIC is the norm is not associated with more CWIC provision.
• Higher perceived behavioral control is not associated with more CWIC provision.

## 1 Introduction

Clinical Work-Integrating Care (CWIC) is a form of clinical healthcare that looks at the interrelationship between paid employment and health [1,2]. When providing CWIC, a healthcare professional takes into consideration both how work-related factors can affect health and how the impact of disease and actions from clinical healthcare professionals can affect work participation [1].

Depending on the patient's context, there are various ways in which CWIC could be provided, but it always starts with asking whether a patient works. Based on the patient's specific needs, CWIC could contain actions like exploring whether work factors cause the patients symptoms. For example, because a patients with asthma might want to know how to reduce the symptoms during working hours. Another action could be to refer a patient to an occupational health physician to support a cancer survivor to return to work, because this patient has difficulties coping with medical complaints and work. These are just some examples of what kind of actions could be taken to provide CWIC [1,3]. Furthermore, not all actions of CWIC will be addressed equally often during a clinical consultation, because it might not be relevant in a certain situation.

Currently CWIC is provided in a wide variety, ranging from assessing a patient's occupation to extensive work-related support [2]. Few guidelines on work participation are available for medical specialists [[4], [5], [6], [7], [8]]. The general lack of guidelines could explain this wide variety [2]. Internationally, the provision of CWIC is still largely driven by regulations, such as within the context of sick notes [2]. Nonetheless, work as a social determinant of health has increasingly gained awareness within clinical healthcare [9]. Patients in previous studies have expressed a need to discuss work with their medical specialists [1,10,11] and to have continuous and supportive care with regard to work participation issues that is tailored to their individual needs [12]. Yet CWIC within specialized medical care (i.e., clinical healthcare provided by medical specialists performed within a hospital setting) is most often only initiated at a patient's request [2,13]—if the topic is not raised by the patient, work participation issues are currently not regularly discussed in specialized medical care [2,14,15]. Furthermore, the work-related advice provided after such requests has been found to be limited and not sufficiently tailored to a patient's situation [[16], [17], [18], [19]] and other medical issues are likely to be prioritized by medical specialists in daily practice [20]. Since maintaining work can be regarded as an important treatment goal in specialized medical care [21], increased provision of CWIC may be valuable. Yet, it is unknown why individual medical specialists do or do not provide CWIC.

For the purpose of this study we define *providing CWIC* as asking whether a patient is working and considering together with the patient through a few exploratory questions whether further attention to work participation issues during the consultation or any follow-up appointments may be needed [1,2]. In some cases, patients may have more complex work-related questions that require referral to or cooperation with occupational healthcare professionals [1,2,22,23].

We applied the reasoned action approach [[24], [25], [26]] to reveal the underlying determinants that influence the frequency at which medical specialists provide CWIC [[24], [25], [26]]. The reasoned action approach was chosen because it is a comprehensive and empirically supported framework for understanding and predicting human behavior [[27], [28], [29]]. This model states that predictors for performing certain actions are the *attitude* (e.g., finding it important to provide CWIC to patients), *perceived norm* (e.g., the social pressure felt because colleagues provide CWIC and consider it part of standard care), and *perceived behavioral control* (e.g., perception one has enough knowledge, skills and capacity to provide CWIC). The actions a person ultimately takes are also influenced by that person's actual control over the situation (e.g., a person's actual skills or knowledge about providing CWIC and environmental factors such as time during a consultation). As such, our hypothesis was that having a favorable attitude towards CWIC, a perception that CWIC is the norm, and a high perceived behavioral control for integrating work participation issues into clinical practice would lead a medical specialist to include CWIC in patient consultations.

To our knowledge this is the first study to examine CWIC provision or a similar construct such as work-oriented care, work-related support, or work-focused healthcare [[30], [31], [32]] using a reasoned action approach. The aim of this study was to examine the association of attitude, perceived norm, and perceived behavioral control with frequency with which medical specialists provide CWIC.

## 2 Methods

### 2.1 Study design

The data from this research was obtained as part of a larger study [3]. A cross-sectional survey was distributed among Dutch medical specialists between February–June 2022. We did not assume any time factor effects. The survey was disseminated via the newsletters of several medical specialist associations, through snowballing, and with a social media campaign on LinkedIn. Medical specialists or physicians in postgraduate training to become specialists were eligible if they (i) practiced in secondary or tertiary clinical healthcare, (ii) were involved in direct patient consultation, and (iii) treated patients of working age (18–67 years). This survey was based on a previous study that explored the patient perspectives and needs for CWIC [1] and a scoping review that explored the extent and nature of CWIC [2], and consisted of several clusters. The three clusters relevant for the current research were: 1) background characteristics, 2) frequency of providing CWIC in the current practice, and 3) attitudinal beliefs, normative beliefs, and control beliefs about CWIC. A pilot test was performed with ten medical specialists to determine the face validity of the questions before dissemination of the survey. Minor revisions of the questions were made based on the feedback of the respondents. No additional validity evidence was obtained beyond the pilot test. We used STOBE checklist for cross-sectional studies for reporting this mixed-methods study [33].

### 2.2 Measures

For this study, we were primarily interested in the underlying determinants of providing CWIC as an overarching way of approaching a patient across specializations, rather than the individual elements, which may not be relevant in every context. Frequency of providing CWIC was the dependent variable in this study. The construct of providing CWIC was operationalized as performing the following actions within clinical medical care: *asking about work; taking a short work history; taking initiative to discuss the topic of work with a patient; and addressing the topics of work causing disease, disease affecting work, treatment affecting work, and laws and regulations regarding disease and work*. These actions were chosen since they reflect what a medical specialist does on their own initiative, excluding the provision of CWIC initiated by others such as the topic ‘work’ being raised by the patient. To assess the frequency of CWIC provision we used a 5-point Likert scale (0 = never, 4 = always) with seven self-developed questions (Supplementary file A). Three questions asked the participants to rate their actions (e.g., ‘how often do you ask whether your patient works?’) and four questions asked them to rate how often different work-related topics were discussed (e.g., ‘how often do you discuss the influence of work on disease?’). For these seven questions, a sum score was calculated. This score was subsequently converted into a sum score between 0 and 100 to facilitate comparison with other sum scores. Higher scores indicate that a specialist self-reported providing CWIC more frequently. Cronbach's Alpha of the construct providing CWIC was 0.88.

The independent variables of a medical specialist's attitude, perceived norm, and perceived behavioral control related to providing CWIC were examined with eight statements about providing CWIC oneself (e.g., ‘I consider it important to discuss work with my patients’), eight statements concerning perceived norms about CWIC (e.g., ‘my colleagues consider it important to discuss work with their patients’), and nine statements concerning perceived behavioral control about CWIC (e.g., ‘if I receive a question from my patient about his or her work, I have sufficient knowledge to advice on it’). All statements could be answered on a five-point Likert scale (1 = strongly disagree, 5 = strongly agree). Attitude, perceived norm, and perceived behavioral control were then calculated as three sum scores of items. These scores were also converted into a sum score between 0 and 100. Higher scores indicate that a specialist has a favorable attitude, a perception that CWIC is the norm, and a high perceived control towards providing CWIC. Cronbach's Alpha of the constructs attitude, perceived norm, and perceived behavioral control were respectively 0.81, 0.91, and 0.69.

The following background characteristics were assessed with multiple choice questions: medical specialization (e.g., dermatology, orthopedic surgery, rehabilitation medicine); gender of the specialist; years of medical work experience (i.e., <5, 5–10, 10–20, and > 20); the specialist's estimation of the percentage of consultations with working patients (i.e., 0–20 %, 20–40 %, 40–60 %, 60–80 %, 80–100 %, and unknown); and type of patients (i.e., mostly chronic or mostly acute conditions). Since some medical specialties included a small number (*n* < 10) of individuals, we categorized the specialties into three groups (i.e., surgical, non-surgical, and rehabilitation specialists) to perform the analysis.

### 2.3 Analysis

Background characteristics were analyzed with descriptive statistics. Univariable and multivariable regression analysis were used to investigate if the variables attitude, perceived norm, and perceived behavioral control were associated with the frequency to which medical specialists provide CWIC in clinical practice. First, three univariable analyses were performed (without considering confounders) in which the frequency of providing CWIC was the dependent variable and attitude, perceived norm, and perceived behavioral control were the independent variables. Next, multivariable analysis was performed without considering confounders using the same dependent and independent variables. Third, background factors (i.e., specialization, gender, experience, percentage of working patients, type of patients) were added as confounders to the multivariable regression model. These background factors were selected as confounders because, based on our interview findings conducted after the survey, we considered them to potentially influence both the dependent variable (*providing CWIC*) and the independent variables (*attitude*, *perceived norm*, and *perceived behavioral control about providing CWIC*) [3]. Finally, a post hoc analysis was performed which excluded rehabilitation specialists from the above-mentioned analyses. This exclusion was made because, given that by definition the purpose of rehabilitation medicine is to improve societal participation (including work participation) [34], rehabilitation specialists might have different attitudes, perceived norm, and perceived behavioral control towards providing CWIC compared to other medical specialists.

Due to the exploratory nature of this research and the unavailability of previous data, we could not perform a sample size calculation prior to the data collection nor a post-hoc power analysis. Participants with missing data were excluded from the analysis.

### 2.4 Ethical approval

The study adhered to the general principles of research ethics outlined in the 2013 World Medical Association Declaration of Helsinki [35]. Our sample consisted of medical specialists questioned about their profession and because this type of inquiry is not subject to the Dutch law of medical scientific research (WMO) [36], ethical approval was not required. All participants volunteered and informed consent was considered implicit upon their submission of the online survey.

## 3 Results

In total, 160 surveys were returned (response rate is unknown due to sampling method) of which 87 % completed it. The sample consisted of 12 surgical specialists (8 %), 113 non-surgical specialists (71 %), and 35 rehabilitation specialists (22 %). The sample consisted of slightly more experienced specialists as opposed to less experienced specialists (56 % with >10 years of experience versus 44 % with <10 years of experience). Two thirds of the sample consisted of female specialists (64 %). Most specialists estimated that about half of their patient population worked, and specialists indicated that they mainly treated patients with chronic conditions (rather than acute conditions) (Table 1). The mean sum scores of providing CWIC, attitudes, perceived norm, and perceived behavioral control are listed in Table 1.

**Table 1:** Background characteristics of the sample and sum scores of the frequency of providing CWIC, attitude, perceived norm and perceived behavioral control.

Background characteristic	Frequency (%)
Total	160 (100)
Medical specialty	
Surgical specialties	12 (7.5)
Non-surgical specialties other than rehabilitation	113 (70.6)
Rehabilitation specialties	35 (21.9)
Years of experience	
In medical specialist training	18 (11.3)
<5 years	20 (12.5)
5–10 years	32 (20.0)
10–20 years	41 (25.6)
>20 years	49 (30.6)
Gender	
Female	103 (64.4)
Male	57 (35.6)
Estimation of working patients	
0–20 %	3 (1.9)
20–40 %	25 (15.6)
40–60 %	80 (50.0)
60–80 %	48 (30.0)
80–100 %	4 (2.5)
Treats mostly patients with chronic conditions^1^	140 (87.5)
Treats mostly patients with acute conditions^1^	36 (22.5)

Sum score (0−100)^2^	Mean (SD)
Frequency of providing CWIC (*n* = 160)	59.84 (18.02)
Attitude (*n* = 153)	73.86 (13.01)
Perceived norm (*n* = 146)	61.30 (15.79)
Perceived behavioral control (*n* = 139)	59.69 (12.05)

Abbreviates: SD, standard deviation.

NOTE: these data are also presented as part of our larger study [37].

1 Asked as two separate questions, thus combinations are possible.

2 Higher scores (0–100) indicate that a specialist provides CWIC more frequently, or that a specialist has a favorable attitude, a perception that CWIC is the norm, and a high perceived control towards providing CWIC.

Results of the univariable regression analyses showed that a more favorable attitude (B = 0.84, SE = 0.09, *p* < .01), a more affirmative perceived norm (B = 0.62, SE = 0.08, p < .01), and higher perceived behavioral control (B = 0.68, SE = 0.11, p < .01) were associated with a higher frequency of providing CWIC. The explained variance (R^2^) of attitude, perceived norm, and perceived behavioral control were respectively 0.38, 0.30, and 0.21.

Results of the multivariable regression analyses showed that a more favorable attitude and a perception that CWIC is the norm were significantly associated with a higher frequency of providing CWIC (B = 0.58, SE = 0.12, *p* < .01; and B = 0.29, SE = 0.09, p < .01, respectively). Higher perceived behavioral control was not significantly associated with a higher frequency of providing CWIC (B = 0.09, SE 0.13, *p* = .49). The explained variance of the model was 0.43.

After adjustment for confounders, the results of the multivariable regression analyses showed that only a more favorable attitude was significantly associated with a higher frequency of providing CWIC (B = 0.56, SE 0.12, *p* < .01), whereas perceived norm and perceived behavioral control were not (B = 0.03, SE = 0.10, *p* = .74; and B = −0.02, SE = 0.13, *p* = .85, respectively) (Table 2). The explained variance of the model was 0.57.

**Table 2:** Regression analyses of the association of attitude, perceived norm and perceived behavioral control with proving CWIC.

Variable	Univariable		Multivariable		Adjusted multivariable	
	B (SE)	p	B (SE)	p	B (SE)	p
Attitude	0.837 (0.087)⁎	<0.01	0.575 (0.124)⁎	<0.01	0.556 (0.118)⁎	<0.01
Perceived norm	0.620 (0.078)⁎	<0.01	0.293 (0.095)⁎	<0.01	0.034 (0.103)	0.74
Perceived behavioral control	0.684 (0.114)⁎	<0.01	0.088 (0.128)	0.49	−0.024 (0.125)	0.85
Gender						
Female					0	
Male					0.073 (2.478)	0.98
Medical specialty						
Rehabilitation medicine					0	
Non-surgical specialties					−16.662 (3.737)⁎	<0.01
Surgical specialties					−15.484 (5.258)⁎	<0.01
Physicians work experience						
>20 years					0	
10–20 years					−0.054 (3.176)	0.99
5–10 years					−1.988 (3.287)	0.55
<5 years					−8.295 (3.822)⁎	0.03
Resident in training to become medical specialist					−8.407 (4.211)⁎	0.05
Physicians estimated percentage of working patient population						
80–100 %					0	
60–80 %					3.281 (7.218)	0.65
40–60 %					0.417 (7.236)	0.95
20–40 %					−2.710 (7.607)	0.72
0–20 %					−4.085 (12.025)	0.74
Treats mostly patients with chronic conditions					−1.728 (3.470)	0.62
Treats mostly patients with acute conditions					1.035 (2.738)	0.71

⁎ Significant.

Post-hoc analysis showed that the results were robust whether or not rehabilitation specialists were included (Supplementary file B).

## 4 Discussion and conclusion

### 4.1 Discussion

The aim of this study was to examine the association of attitude, perceived norm, and perceived behavioral control with the frequency that medical specialists provide CWIC. The analysis revealed that having a favorable attitude towards CWIC is the only determinant significantly associated with a higher frequency of CWIC provision by medical specialists. Neither a higher perception that CWIC is the norm nor higher perceived behavioral control were associated with providing CWIC more often. This finding rejects our hypothesis that all three factors would be positively associated with providing CWIC.

Differentiating between the construct *attitude*, *perceived norm*, and *perceived behavioral control* is important to understand why a person performs a certain action, because this provides actionable insight to influence that behavior [25,38]. Depending on the population and the context in which an action is performed, this could be entirely driven by one of these constructs or a combination of attitudinal, normative or behavioral control considerations [25,38,39]. In our study we found that *providing CWIC* is driven by attitudinal considerations only. Medical specialists that self-reported to provide CWIC more frequently had a favorable attitude that reflected a positive view of addressing and integrating work into medical treatment, recognizing it as a relevant component of healthcare within the scope of the medical specialist's professional responsibilities.

It is important to note that in this study *providing* CWIC refers to the first steps of CWIC provision (i.e., asking whether a patient is working and probing with a few exploratory questions). We suspect that for some sequential actions of providing CWIC other determinants could be more dominant besides attitude. For example, the action ‘referring a patient to an occupational physician’ which will only come to play after initially discussing work with a patient. In the Netherlands, contacting the patients' occupational health physician is often found difficult, because, among others, the patient does not know who this physician is or cannot relay any contact information [3,22]. Therefore, is seems logical that perceived and actual control for this action in providing CWIC are expected to be more prominent determinants in the association.

The lack of a significant association between perceived norm and the frequency of providing CWIC could have resulted from the fact that social norm encompass different types of norm [40]. In our survey the questions were designed to assess perceived norm by presenting statements that reflected on the specialists' beliefs about their colleagues' views on the importance of providing CWIC (i.e., *descriptive norms*). Respondents being unsure about their colleagues' behavior or beliefs on the subject may have led to neutral responses to the perceived norm questions and the resulting lack of association. However, in interviews we held after the survey as part of the larger mixed methods study to which the current study belongs, rehabilitation specialists indicated that the implementation of new guidelines had influenced their professional norm (i.e., *injunctive norms*) [37]. They explained that these new guidelines had influenced their decision to integrate the topic of work into their current practice more than what they believed other specialists do during consultations. Indeed, clinical practice guidelines are often seen as tools to establish and reinforce social norms within professional groups [41,42]. To improve the process and structure of care, evidence-based clinical guidelines can be effective [43]. Yet, to determine whether this holds true for providing CWIC, injunctive norms should be measured in future research. After data collection for the current study a generic guideline on work participation became available to all Dutch medical specialists [44].

The lack of association between perceived behavioral control and the frequency of providing CWIC might be explained by the relationship that perceived control has with actual control according to the reasoned action approach [24,25]. In the context of providing CWIC, actual control is mostly determined by environmental factors and the skills or knowledge of a specialist regarding work-related topics. The environmental factors *setting* and *patient population* were included in our survey as background factors, since it is understandable that in some situations (such as in an acute or life-threatening situation) work-related issues would not be prioritized in a one-time encounter whether or not a patient is within working age. Other environmental factors such as *consultation time* and *communication with occupational healthcare* were assessed as perceived behavioral control, as were the specialists' skills and knowledge. Specialists were asked about their confidence in their skills, knowledge, and time for providing CWIC, and about their confidence in communication with occupational healthcare. Still, although the perception of having enough time or knowledge is a good indicator of someone capacities and abilities, it does not necessarily reflect the actual time or knowledge a person has. Indeed, in daily practice medical specialists often experience heavy workloads that impact their ability to provide CWIC [30]. Furthermore, hospital-wide structural arrangements to make referrals or coordinate between clinical and occupational healthcare possible are lacking [22,[45], [46], [47]] and medical education about work and health is limited [[48], [49], [50], [51]]. This is why other studies have highlighted the importance of professional skills training to provide CWIC and emphasized the need for improved structural arrangements, such as time, financial support, and work-related expertise within hospitals [26,30].

At the time our survey was conducted, COVID-19 measures had just been lifted. However, we did not consider this to have affected our findings. This was evident from the interviews conducted after the survey, during which the specialists did not mention COVID-19 as a factor influencing whether they provided CWIC or not [3]. However, there was an unequal distribution of medical specialties in our sample when compared to the registered medical specialists in the Netherlands [52]. Specifically, surgical specialists were underrepresented, and rehabilitation specialists were overrepresented. This might indicate selection bias. The post hoc analysis we performed excluding rehabilitation specialists showed our results to be robust with or without this group. However, our findings need to be interpreted with care when extrapolating them for surgical specialists, since perceptions of non-surgical specialists may not be generalizable. Furthermore, there was a notable drop-out from the survey, possibly indicating attrition bias. As a consequence, the findings may be positively biased and less generalizable to the broader target population, particularly those with lower levels of affinity with the topic.

No validated questionnaire was available for measuring the provision of CWIC, and therefore we created our own survey. This survey was pilot tested to assess its face validity. Although we belief we measured all important aspects of providing CWIC and attitudes, perceived norms and perceived behavioral control about providing CWIC by using our previous research on the topic [1,2], potentially we could have missed an aspect. The internal consistency (Cronbach's Alpha) of the constructs was acceptable for perceived behavioral control, good for the frequency of providing CWIC and attitude, and excellent for perceived norm. The internal consistency of perceived behavioral control was relatively low. This was probably caused by the fact that five out of nine statements to measure perceived control had to be reversed due to negative phrasing of the questions. This may have affected the lack of association found, because it confuses respondents to switch back and forth between a negative and positive interpretive frame [53]. Furthermore, although it is logical to reverse the phrase “I have” versus “I do not have”, the reasons for providing CWIC are not necessarily the direct opposites of the reasons for not providing CWIC [25]. Although the internal consistency for the construct providing CWIC was good, it is important to note that it was based on self-reported behavior regarding how frequently these actions were performed. Since the behavior was self-reported, specialists with a more positive attitude may have overestimated how often they provide CWIC, potentially inflating the association found between attitude and providing CWIC.

### 4.2 Innovations

To our knowledge this is the first study to examine CWIC using a reasoned action approach. The reasoned action approach and its predecessor the theory of planned behavior (i.e., ASE-model) are commonly used to understand why people take certain actions and as such aid in detecting the specific determinants that need to be targeted in a behavioral change program [54]. Previous programs that aimed to integrate work into specialized medical care used the ASE-model to address healthcare providers' attitudes, social norm, and self-efficacy in providing based on qualitative findings [26,30]. However, to our knowledge this study is the first to use quantitative data to establish that a more favorable attitude is indeed associated with providing CWIC more often.

To implement CWIC as an innovation in clinical practice, it will be important to increase the number of medical specialists with a favorable attitude towards providing CWIC. A positive view on addressing and integrating work into medical treatment, and recognizing it as a relevant component of good care within the scope of the medical specialist's professional responsibilities is key to the further integration of CWIC into routine practice. This could be achieved by addressing medical specialists' attitudes by raising awareness about the importance of CWIC, for example, through targeted campaigns emphasizing the importance of discussing work with patients and its potential benefits for medical treatment. This might be accomplished by involving medical specialists in the development of programs aimed at integrating work participation issues into specialized medical care and by designing medical education and practical tools for the providers [26,30]. Furthermore, it might aid to maintain a favorable attitude when structural arrangements are made that simplify referrals and coordination between clinical and occupational healthcare [30,55,56]. It is recommended that when developing a program this aligns with the professional norm of medical specialists, such as by being expressed in guidelines [7,8,44].

### 4.3 Conclusion

Medical specialists who express a favorable attitude towards the topic of work are more inclined to provide CWIC. It is recommended that specialists' attitude towards providing CWIC be addressed in future initiatives aimed at integrating CWIC in specialized medical care to improve care for patients who work.

## CRediT authorship contribution statement

**Authors: Lana Kluit:** Writing – original draft, Validation, Resources, Project administration, Methodology, Investigation, Formal analysis, Data curation, Conceptualization. **Annechien Beumer:** Writing – original draft, Supervision, Resources, Methodology, Conceptualization. **Coen A.M. van Bennekom:** Writing – original draft, Supervision, Resources, Methodology, Conceptualization. **Angela G.E.M. de Boer:** Writing – original draft, Validation, Supervision, Methodology, Formal analysis, Conceptualization. **Astrid de Wind:** Writing – original draft, Validation, Supervision, Methodology, Formal analysis, Conceptualization.

## Funding sources

This research did not receive any specific grant from funding agencies in the public, commercial, or not-for-profit sectors.

## Declaration of competing interest

The authors declare that they have no known competing financial interests or personal relationships that could have appeared to influence the work reported in this paper.

## Data Availability

The datasets generated and analyzed during the current study are not publicly available but are available from the corresponding author on request.

## Supplementary data

Supplementary material 1: Supplementary file A: survey questions Image 1

Supplementary material 2: Supplementary file B Post-hoc analysis Image 2
