# Supplementary material for: The association between attitude, perceived norm, and perceived behavioral control with the provision of Clinical Work-Integrating Care: A reasoned action approach
Source: PEC Innov. 2025 Jun 27;7:100416. doi: 10.1016/j.pecinn.2025.100416 (PMC12273432; doi:10.1016/j.pecinn.2025.100416)
Supplement: Supplementary file 1 — Supplementary material 1: Supplementary file A: survey questions [file mmc1.docx]

## Supplementary file A Survey questions to measure providing CWIC, attitude, perceived norm and perceived behavioral control

### Questions to assess the extent of Providing CWIC

*Questions were asked on a 5-point Likert scale (0 = never, 4 = always)*

Can you please indicate how often this occurs in the questions below:

1. How often do you ask if your patient is working?
2. How often do you take a work history*?

How often are the topics below discussed during your contact with patients:

1. Influence of work on illness (as a cause of illness or worsening of symptoms)
2. Influence of illness on work ability
3. Influence of treatment on work ability
4. Questions about legislation and regulations regarding absenteeism from work or disability due to illness

On whose initiative do you discuss the topic of ‘work’ with your patients?

1. On my own initiative

*A work history includes questions such as: ‘Are you currently employed?’ ‘What kind of work do you do?’ ‘Are you currently out of work?’ ‘And which health complaints play a role in this?’ ‘Do you work in shifts?’ ‘Are your health complaints lessened when you don’t work, for example, at the weekend or during the holidays?’ ‘Do you have work problems?’ ‘Are you in contact with an occupational physician?’

### Questions to assess the medical specialists’ attitude, perceived norm, and perceived behavioral control about providing CWIC

*Questions were asked on a 5-point Likert scale (1= strongly disagree, 5= strongly agree)*

#### Attitude about providing CWIC

To what extent do you agree with the following statements?

1. I consider it important to discuss work with my patients.
2. I consider that I should pay attention to work during the treatment process.
3. I consider it important to view my patients as a whole, of which work is a part.
4. I consider work an important outcome measure in treatment.
5. To practice my specialty, I consider that asking questions from the occupational history can be an important addition to providing good care (for example, for accurate diagnostics).
6. If my patient has ‘being able to work’ as a goal, I consider it important to take this into account in the treatment plan.
7. I consider that giving advice about work is not a task of a medical specialist.
8. If I receive a question from a patient about his or her work, I consider that I am not allowed to make a statement about it.

#### Perceived norm about providing CWIC

To what extent do you agree with the following statements?^*^

1. My colleagues consider it important to discuss work with patients.
2. My colleagues pay attention to work participation during the treatment process.
3. My colleagues consider it important to view their patients as a whole, of which work is a part.
4. My colleagues consider work an important outcome measure in a successful treatment.
5. Within my specialty, my colleagues consider taking an occupational history important for providing good care (for example, for accurate diagnostics).
6. If a patient has ‘being able to work’ as a goal, my colleagues consider it important to take this into account in the treatment plan.
7. My colleagues consider that giving advice about work is not a task of medical specialists.
8. My colleagues regularly consult with the occupational health physician of their patient.

^*^‘My colleagues’ refers to medical specialists with whom you work and who treat a similar patient population as yourself

#### Perceived behavioral control about providing CWIC

To what extent do you agree with the following statements?

1. If I receive a question from my patient about his or her work, I have sufficient knowledge to advise on it.
2. I have enough information available (such as guidelines) to answer work-related questions.
3. I know which tasks an occupational health physician performs.
4. I know which tasks an insurance physician (from the UWV) performs.
5. I have no time to deal with work-related questions from my patients.
6. I am afraid of legal consequences when giving advice about work.
7. I do not contact an occupational health or insurance physician because of privacy legislation.
8. I do not answer questions from the occupational health physician because I receive no financial compensation for this.
9. I do not answer questions from the occupational health physician because I never receive feedback on what is done with the information.
