# Supplementary material for: The association between attitude, perceived norm, and perceived behavioral control with the provision of Clinical Work-Integrating Care: A reasoned action approach
Source: PEC Innov. 2025 Jun 27;7:100416. doi: 10.1016/j.pecinn.2025.100416 (PMC12273432; doi:10.1016/j.pecinn.2025.100416)
Supplement: Supplementary file 2 — Supplementary material 2: Supplementary file B Post-hoc analysis [file mmc2.docx]

## Supplementary file B Post-hoc analysis

The sum score of providing CWIC, attitude, perceived norm and perceived control excluding the responses of rehabilitation specialists are listed in Table 1.

Table 1 Sum scores without rehabilitation specialists

| **Sum score (0-100)** | **Number of responses** | **Mean (SD)** |
| --- | --- | --- |
| Extent of providing CWIC | 125 | 54.43 (14.45) |
| Attitude | 119 | 70.59 (11.96) |
| Perceived norm | 112 | 55.66 (11.68) |
| Perceived behavioral control | 105 | 56.42 (10.22) |
| Abbreviates: SD, standard deviation | | |

Results of the univariable regression analyses showed that a more favorable attitude (B = 0.52, SE = 0.09, p <.01), a more affirmative perceived norm (B = 0.30, SE = 0.12, p = 0.01), and a higher perceived behavioral control (B = 0.40, SE = 0.14, p <.01) were associated with a higher frequency of providing CWIC. The explained variance (R^2^) of attitude, perceived norm, and perceived behavioral control were respectively 0.23, 0.06, and 0.07.

Results of the multivariable regression analysis without confounders showed that a more favorable attitude was significantly associated with a higher extent of providing CWIC (B = 0.56, SE = 0.13, p <.01). Perceived norm and control were not significantly associated with providing CWIC (B = 0.07, SE 0.12, p = 0.59; and B = 0.04, SE = 0.15, p = 0.79, respectively). The explained variance of the model was 0.24.

After adjustment for medical specialty, gender, work experience, estimated percentage of working patient population, and type of patients, only an favorable attitude was significantly associated with a higher extent of providing CWIC (B = 0.58, SE 0.13, p <.01), and perceived norm and perceived control were not (B = 0.01, SE = 0.12, p = 0.95; and B = 0.09, SE = 0.16, p = 0.56, respectively). The explained variance of the model was 0.36 (Table 2).

Table 2 Regression analysis of the association of attitude, perceived norm and perceived control with proving CWIC

| **Variable** | | **Univariable** | | **Multivariable** | | **Adjusted multivariable** | |  |
| --- | --- | --- | --- | --- | --- | --- | --- | --- |
|  |  | **B (SE)** | **p** | **B (SE)** | **p** | **B (SE)** | **p** |  |
| Attitude | | 0.519 (0.087)* | <.01 | 0.563 (0.130)* | <.01 | 0.586 (0.134)* | <.01 |  |
| Perceived norm | | 0.299 (0.118)* | .01 | 0.066 (0.123) | 0.59 | 0.008 (0.122) | 0.95 |  |
| Perceived behavioral control | | 0.399 (0.143)* | <.01 | 0.040 (0.153) | 0.79 | 0.094 (0.159) | 0.56 |  |
| Gender | |  |  |  |  |  |  |  |
|  | Female |  |  |  |  | 0 |  |  |
|  | Male |  |  |  |  | -1.348 (2.924) | 0.68 |  |
| Medical specialty | |  |  |  |  |  |  |  |
|  | Rehabilitation medicine |  |  |  |  | 0 |  | |
|  | Non-surgical specialties |  |  |  |  | -16.662 (3.737)* | <.01 |  |
|  | Surgical specialties |  |  |  |  | -15.484 (5.258)* | <.01 |  |
| Physicians work experience | |  |  |  |  |  |  |  |
|  | >20 years |  |  |  |  | 0 |  |  |
|  | 10-20 years |  |  |  |  | -1.494 (3.648) | 0.68 |  |
|  | 5-10 years |  |  |  |  | -3.586 (4.040) | 0.38 |  |
|  | <5 years |  |  |  |  | -12.092 (4.732)* | 0.01 |  |
|  | Resident in training to become medical specialist |  |  |  |  | -13.036 (5.029)* | 0.01 |  |
| Physicians estimated percentage of working patient population | |  |  |  |  |  |  |  |
|  | 80-100% |  |  |  |  | 0 |  |  |
|  | 60-80% |  |  |  |  | 1.473 (14.258) | 0.92 |  |
|  | 40-60% |  |  |  |  | -1.516 (14.085) | 0.92 |  |
|  | 20-40% |  |  |  |  | -6.570 (14.467) | 0.65 |  |
|  | 0-20% |  |  |  |  | -3.795 (17.355) | 0.83 |  |
| Treats mostly patients with chronic conditions | |  |  |  |  | -1.735 (4.099) | 0.67 |  |
| Treats mostly patients with acute conditions | |  |  |  |  | 0.157 (3.343) | 0.96 |  |
| *Significant | | | | | | | |  |
